# Supplementary material for: Comparative study of enriched dopaminergic neurons from siblings with Gaucher disease discordant for parkinsonism
Source: bioRxiv. 2024 Feb 28:2024.02.25.581985. Preprint. [Version 1] doi: 10.1101/2024.02.25.581985 (PMC10962709; doi:10.1101/2024.02.25.581985)
Supplement: Supplement 1 [file NIHPP2024.02.25.581985v1-supplement-1.pdf]

bioRxiv preprint doi: https://doi.org/10.1101/2024.02.25.581985; this version posted February 28, 2024. The copyright holder for this preprint (which was not certified by peer review) is the author/funder. This article is a US Government work. It is not subject to copyright under 17 USC 105 and is also made available for use under a CC0 license.

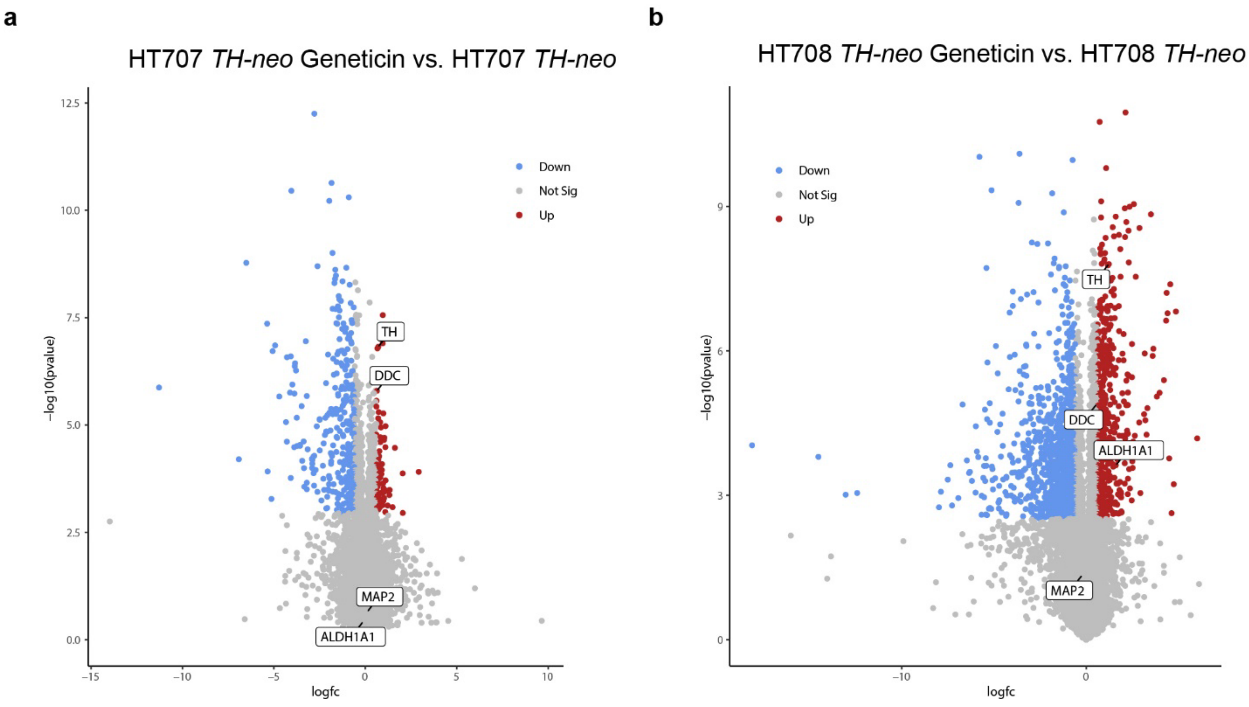

Supplementary Figure 1

Volcano plots showing enrichment of dopaminergic markers for HT707 *TH-neo* (a) and HT708 *TH-neo* (b) after treatment with geneticin.

Supplementary Table 1 Primers used

Set	
1 Forward	5'-AGCCCTCTAGCCTCATCCTC
1 Reverse	5'-ACATCCCCTGCTTGTTTCAACAG
2 Forward	5'-CCCTCAGACCCTTTTAGTCAGTGTG
2 Reverse	5'-CCAGCCAGCACAGTTTGTGAC

Supplementary Table 2 Antibodies used in this study

**Antibody**	**Company**	**Catalogue number**	**Application**
GAPDH	abcam	Ab9485	WB (1:2000)
GCase	Abnova	H00002629-M01	WB (1:50)
TH	Pel-Freeze	P60101-150	IF (1:400), FC (1:400), WB (1:50)
FoxA2	Neuromics	RA34008	IF (1:500)
Neo II	Sigma	06-747	IF (1:500)
